# Succinct workflows for circulating tumor cells after enrichment: From systematic counting to mutational profiling

**DOI:** 10.1371/journal.pone.0177276

**Published:** 2017-05-08

**Authors:** Victor Chun-Lam Wong, Josephine Mun-Yee Ko, Chi-Tat Lam, Maria Li Lung

**Affiliations:** 1 OncoSeek Limited, Hong Kong Science and Technology Parks, Hong Kong Special Administrative Region; 2 Department of Clinical Oncology, University of Hong Kong, Hong Kong Special Administrative Region; The Ohio State University, UNITED STATES

## Abstract

**Purpose:**

This study aims to establish a highly adaptable workflow downstream of microfluidic enrichment for facilitating systematic CTC enumeration and genetic discovery.

**Methods:**

To facilitate CTC enumeration, we established a CK/EPCAM-combined immunostaining strategy and an automated CTC analytical pipeline using an open-source image analyzer. By virtue of this workflow, we conducted a pilot study of 56 cancer patients and 21 healthy individuals using a high-throughput spiral microfluidic chip system. To facilitate genetic discovery of somatic mutations in CTCs, we integrated the CTC enumeration into next-generation sequencing and established a straightforward amplicon library comprising diversifier random sequences to sequence CTC samples.

**Results:**

The CTC staining and enumeration workflow achieved 80.4% sensitivity and 85.7% specificity (AUC = 0.87, *p* = 0.004, power = 0.985), as evaluated by ROC analysis. Univariate and multivariate analysis verified that the CTC (CK/EpCAM^+^CD45^−^), but not other cell populations, is a significant and independent biomarker for cancer patients (*p* < 0.01). Serial CTC monitoring of the patients revealed reduction in CTC numbers after treatments, suggesting its clinical utility in pharmacodynamic studies. Deep sequencing of CTC samples revealed somatic mutations in *TP53* and *ESR1*.

**Conclusions:**

The significance of this report is to demonstrate a systematic and adaptable workflow to bridge the gap between the microfluidic enrichment and CTC analyses, which fosters broader applications of CTCs in both clinical settings and academic studies.

## Introduction

Circulating tumor cell (CTC) research has been under the spotlight for non-invasive cancer monitoring [1]. In an era of precision medicine, it is anticipated to guide the selection of effective treatment regimens for patients. Indisputably, the rapid advances in bioengineering have propelled the CTC field. A burgeoning number of compelling microfluidic technologies are emerging to enrich the extremely rare CTC population from billions of blood cells. What follows, importantly, is a streamlined workflow for downstream systematic and unbiased CTC analysis for CTC enumeration and genetic discovery. For CTC identification and enumeration, immuno-detection of antigens on CTCs is by far the most commonly accepted approach. Cytokeratin and EpCAM are surrogate CTC markers widely used in the field [2–4] due to their expression in epithelial cancer cells and their clinical relevance [5–7]. However, with usually only one CTC occurring per 10^7^ WBCs, it remains challenging to identify and count the rare CTCs in the background of white blood cells (WBCs). Therefore, a detailed and validated protocol for the downstream CTC identification, enumeration, and visualization is essentially needed. In some reports, CTCs were identified and counted manually. Manual cell counting is tedious, time-consuming, and subject to bias, which significantly impedes the efforts to achieve clinical utility of CTC analyses. Therefore, a systematic and reliable CTC counting workflow is imperative to obtain high-quality results and promote the applications of CTCs for translational use. This study aims to demonstrate and validate a highly adaptable CTC counting pipeline for systematic CTC application.

Besides CTC enumeration, genomic analysis of CTCs provides a window to strategically assess a potentially metastatic population of cancer cells. For precision medicine, most of the current treatment regimens rely on the genetic information from primary tumors [8], rather than those from the metastatic subpopulation of cancer cells. CTCs are considered as the ‘culprits’ of metastasis, which account for about 90% of cancer deaths [9, 10]. Next-generation sequencing (NGS) analysis of CTCs appears to be an effective tool to provide a snapshot of the rapidly evolving mutation landscape of these metastatic ‘culprits’. The approach offers a way to non-invasively monitor the emerging therapeutic gene targets and drug resistance mutations in real-time. With many convincing CTC isolation technologies emerging, a straightforward targeted sequencing workflow for CTCs is especially needed to broaden the application of CTC technology.

To bridge the aforementioned technical gap between microfluidic enrichment and CTC applications, this study aims to (1) illustrate a systematic CTC enumeration pipeline; (2) establish a straightforward sequencing workflow for CTC samples; and (3) validate the enumeration and sequencing workflows with clinical samples.

## Materials and methods

### Patient & blood collection

All patients had been diagnosed with one of the six primary cancers from the colon, lung, breast, stomach, liver, and prostate from July 2015 to October 2016. All blood samples were obtained after written informed consent from patients. The study was approved by the Institutional Review Board of the University of Hong Kong. A total of 56 cancer patients (22 liver, 11 colorectal, 7 lung, 6 gastric, 5 breast, and 5 prostate cancer) and 21 normal healthy donors were recruited. Eight milliliters of peripheral blood samples were collected in the BCT Cell-Free DNA tubes (Streck Inc., USA). For serial cancer monitoring, blood samples were collected one week before and three months after treatments (radiotherapy or targeted therapy—Sorafenib). All samples were processed within 72 hours of collection.

### Sample preparation and CTC isolation

A volume of eight milliliters of blood samples was used for microfluidic enrichment for each run. Prior to CTC isolation using label-free microfluidic equipment (ClearCell^®^ FX1 system, Clearbridge BioMedics, Singapore), blood samples were subjected to RBC lysis. CTCs are isolated from the blood based on cell size. The isolated CTCs in a background of WBCs were immobilized on positively-charged microscope slides and were subsequently identified by immunofluorescence staining. For cell culture, KYSE30 and KYSE270 esophageal cancer cell lines were cultured in RPMI 1640 medium supplemented with 2mM Glutamine and 10% fetal bovine serum [11, 12].

### Immunofluorescence staining

DAPI, Alexa Fluor 555-conjugated pan-CK (Cell Signaling Technology, USA), Alexa Fluor 555-conjugated EpCAM (Cell Signaling Technology, USA) and APC-conjugated CD45 (BD Biosciences, USA) antibodies were used to identify CTCs. The fluorescent dye-conjugated antibodies were added to cell smears and incubated for two hours at room temperature. After staining, slides were mounted in DAPI-containing anti-fade mounting reagent (Thermo Fisher Scientific, USA) and scanned using a Cytation 5 Cell Imaging Multi-Mode Reader (BioTeck, USA). All images were captured under the same conditions. We applied DAPI staining to label DNA for identifying nucleated cells, CK/EpCAM staining to label CTCs, and CD45 staining to label WBCs.

### CTC enumeration pipeline by CellProfiler and CellProfiler Analyst

The CTC enumeration and visualization process using the CellProfiler and CellProfiler Analyst [13, 14] (http://www.cellprofiler.org) are as follows. 1) Primary object identification: nucleated cells (primary objects) were first identified on the images of DNA staining with the "Global threshold strategy" in CellProfiler. This strategy is robust for detecting cell objects in fluorescent images that have a uniform background. 2) Primary object filtering: the sizes of the nuclei were measured. Nuclei with 9 μm to 36 μm in diameter were filtered for further analysis. 3) Shape measurement: "Eccentricity", a ratio of the between-foci distance and the major axis length, was then measured. The values of eccentricity are from zero to one (a value of zero means circular object). The eccentricity represents nuclear circularity, which is used to exclude WBCs (e.g. neutrophils, basophils, monocytes) that have lobular and irregular nuclear shapes. 4) Secondary object identification: secondary objects were created based on the coordinates of primary objects for measuring quantitative features. 5) Mean intensity measurement: "MeanIntensity", the average pixel intensity within an object, was measured from the secondary objects created on CK/EpCAM and CD45 images. 6) Data export: the measurements were exported as an SQLite database file, which was then opened by CellProfiler Analyst. 7) Cell enumeration: by CellProfiler Analyst, cell gating, enumeration, and visualization were done using the built-in filtering and image-viewing tools on the interactive scatterplots showing the mean intensity and the shape of objects. Positive and negative gates for CK/EpCAM and CD45 staining are set for CTC enumeration and visualization. Two staining controls were used for references: a cancer cell line (KYSE-30) stained with CK, EpCAM, and CD45 antibodies and the buffy coat fraction of the same patients were stained with isotype antibodies.

### Library preparation and next-generation sequencing

Outputs from the microfluidic chip were subjected to DNA extraction using the QIAamp DNA Micro Kit (QIAGEN, Germany). In parallel, five hundred WBCs were counted and subjected to DNA extraction. After DNA quantification with Qubit dsDNA HS Assay Kit (Thermo Fisher Scientific, USA), a 2-step PCR library preparation was performed with Q5 High-Fidelity DNA Polymerases (NEB, USA).

The first PCR serves to amplify target exon regions and introduce a 10-bp diversifier sequence and a portion of sequencing adapters (i.e. P5 and P7 adapters) to the amplicon library. The diversifier sequence is a sequence of ten random nucleotides, which was designed to increase the sequence diversity of libraries for accurate cluster identification by Illumina sequencer. The second PCR serves to extend the remaining portion of Illumina Adapter Sequences (Illumina, USA). Primer sequences are listed in S1 and S2 Tables. Libraries were then purified by magnetic SeqCap EZ purification beads (Roche, Switzerland) and then quantified with NEBNext Library Quant Kit (NEB, USA). Libraries were multiplexed in equimolar amounts and sequenced with MiSeq sequencing kit v2, according to Illumina’s guidelines. In general, 12 pM multiplexed library and 5% phiX spike-in were loaded.

### Bioinformatics analysis

For bioinformatics analysis, the sequencing data from MiSeq were processed by Trimmomatic [15] to trim out P5 and P7 adapters and the diversifier sequences. The sequencing reads were aligned to the reference human genome (hg19) by Burrows—Wheeler Aligner (BWA-MEM) [16]. Subsequently, the aligned files were then calibrated with the GATK Indel Realigner tool [17]. Somatic variant calling was then performed with VarScan2 [18], followed by variant annotation with ANNOVAR [19]. To increase the accuracy of variant identification, tumor purity (i.e. CTC purity) estimated from the CTC counting pipeline was input as a parameter to VarScan2 [18] to adjust variant frequency threshold and optimize Fisher’s exact test. The test compares the number of reference-supporting and variant-supporting reads in tumor samples (i.e. CTC output samples) with those of normal samples (i.e. WBC samples).

For sequencing clinical samples, two tubes of blood (8 mL each) were collected from each patient and were allocated to sequencing workflow and counting workflow in parallel. The tumor purity estimated from counting pipeline was input into VarScan2 for somatic mutation analysis. Mutations with a consistent somatic *p* value lower than 0.05 in two technical repeats were called as consensus somatic mutations.

### Statistical analysis and data visualization

Receiver Operating Characteristic (ROC) Curve Analysis for the estimation of area under the curve, sensitivity, and specificity was performed in SPSS 19 (IBM Corporation, Armonk, NY). The estimation of correlation coefficient, the univariate and multivariate analyses, 2-tailed student *t* test, and 95% confidence interval estimation were performed in R environment (version 3.3.1). The calculation of statistical power was done using pwr package in R. The effect size for the 2-tailed student *t* test was estimated by the difference between two means divided by pooled standard deviation. The effect sizes for the univariate and multivariate analyses were estimated based on the odds ratios. For the Kaplan-Meier (KM) survival analysis, data from TCGA and METABRIC were downloaded from cBioPortal (http://www.cbioportal.org/) and then analyzed and visualized in R. Cancer patients harboring mutations in *NRAS*, *ESR1*, *EGFR*, *KRAS*, *BRAF*, or *TP53* were classified as “With mutation(s)” group while cancer patients with no mutations in those genes were assigned to “No mutation” group for KM analysis. The data visualizations in this study were done with ggplot2, plot3D, plotROC, OmicCircos [20], survminer, and venneuler packages in R.

## Results and discussion

### Overview of CTC analytical workflows

**Fig 1:**
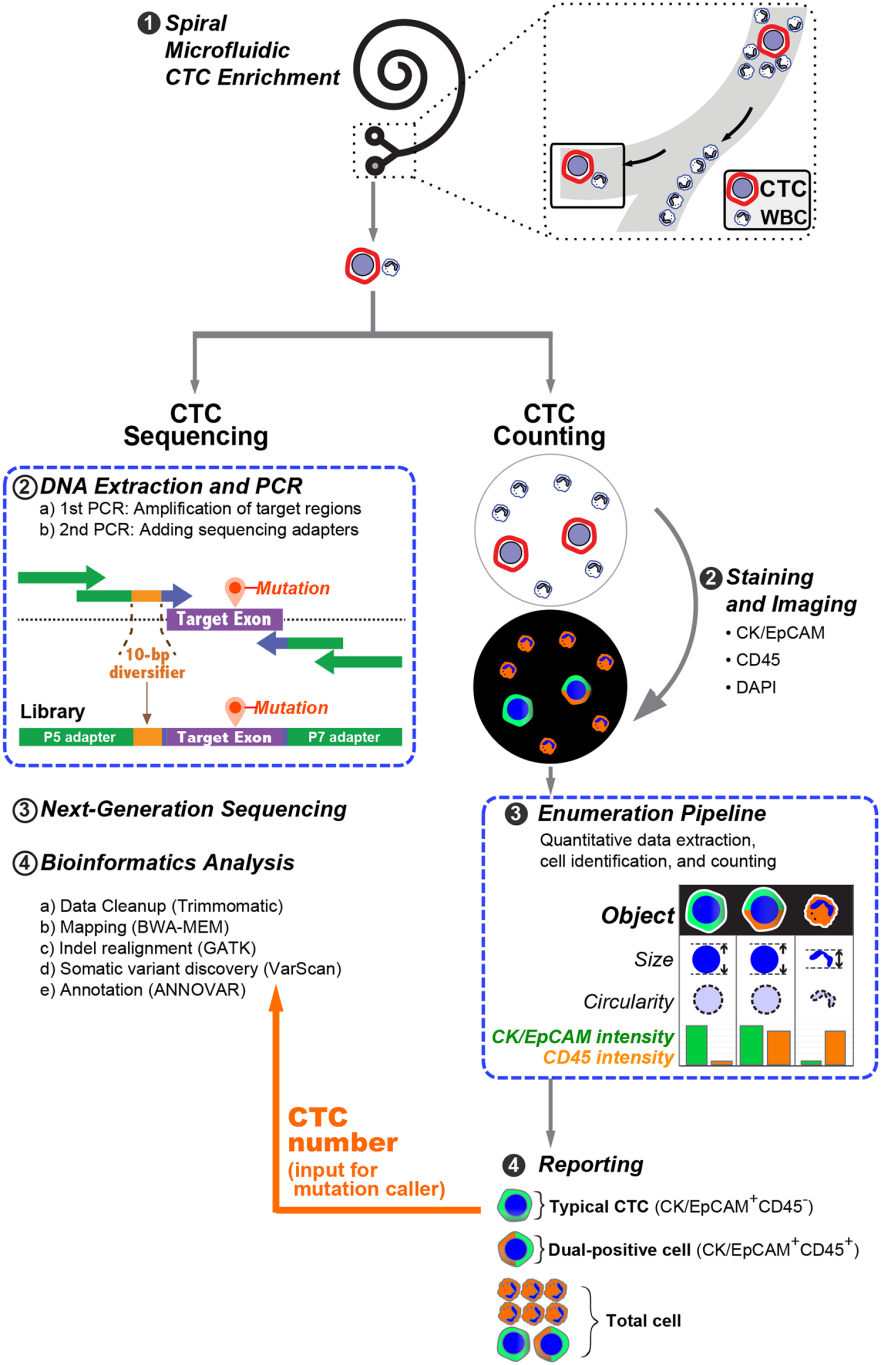
An overview of CTC enrichment, counting, an sequencing workflow.

The spiral microfluidic chip system is one of the highly desirable CTC enrichment methods, which utilizes inherent centrifugal force in the spiral microchannel to deplete WBCs and enrich CTCs based on their cell sizes [21]. The spiral microfluidic chip enriches CTCs by shunting them towards a CTC enrichment outlet. This allows high-throughput and continuous enrichment of CTCs. When compared with immune-mediated CTC capturing systems, the microfluidic platform gives a higher CTC recovery and detection sensitivity [22]. Therefore, we employed this technology to enrich CTCs and to develop a downstream enumeration and sequencing workflow (Fig 1).

In the following sections, we illustrate the collateral workflows for downstream CTC enumeration and sequencing analyses (Fig 1). For the CTC enumeration workflow, we illustrate our CK/EPCAM-combined staining strategy and quantitative image analysis, followed by clinical validation of the workflow in a pilot study of 77 individuals. Next, we demonstrate the importance of the CTC enumeration result in CTC sequencing pipeline. Finally, we show our NGS library design, library preparation protocol, and analytical validation with simulated CTC samples, followed by sequencing of clinical CTC samples.

### CK/EpCAM-combined staining for CTCs

**Fig 2:**
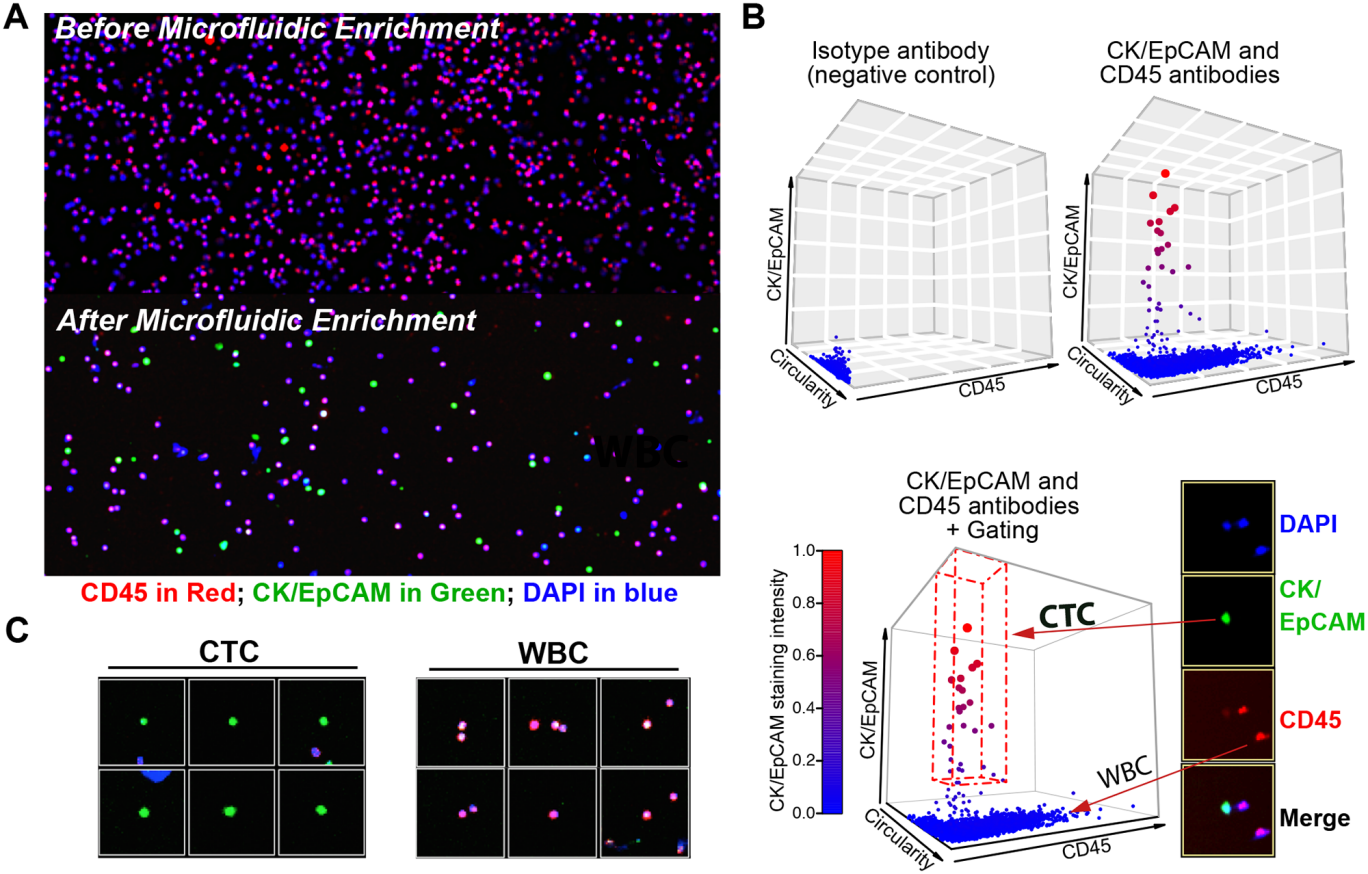
CTC staining, gating, and visualization. (A) Immunofluorescence staining of blood samples before and after microfluidic enrichment. The samples were stained with DAPI (blue), CK/EpCAM (green), and CD45 (red) antibodies. (B) 3D plots showing data extracted from CellProfiler. Cells from buffy coats were stained with isotype control antibodies and served as negative controls. The box with red dashed line indicates gating of CTCs. (C) Individual cell image gallery generated from the enumeration pipeline. CTCs (DAPI^+^CK/EpCAM^+^CD45^−^) and WBCs (DAPI^+^CK/EpCAM^−^CD45^+^) are shown.

To ensure the quality of CTC immunostaining, fluorescence interference should be minimized by avoiding overlap of multiple fluorescence spectra. Therefore, it is important to judiciously utilize the limiting number of available labeling channels. Conventional identification of CTCs requires immunostaining of four markers including CK, EpCAM, CD45, and DAPI [1–4]. Combining different markers of similar function into one channel is a solution to establish a succinct CTC counting workflow to accelerate the process of CTC enumeration. The remaining staining channel allows the opportunity to further understand the CTC biology by staining with additional markers. For instance, cancer stem cell markers (e.g. CD44), EMT markers (e.g. Vimentin), or proliferation markers (e.g. Ki-67) are some interesting biomarkers that merit further exploration. For these reasons, our CTC immunostaining was done by combining CK and EpCAM staining into one fluorescence channel of Alexa Fluor 555 (Fig 2A). The purpose is to establish a succinct staining workflow to expedite the whole staining and enumeration process, as well as allowing the remaining fluorescence channel to be used for other staining studies.

### CTC enumeration and visualization

To establish a systematic CTC enumeration and visualization pipeline, we utilized CellProfiler and CellProfiler Analyst, freely available and versatile image analysis programs developed by the Broad Institute. Automated analysis pipeline was set to measure quantitative features (i.e. mean staining intensities, cell size, and cell shape; see Methods for setting up the pipeline) from fluorescent images. As shown in Fig 2B, a significant population of cells in the CTC microfluidic output express CK/EpCAM proteins. Although the CK/EpCAM expressions of these cells are heterogeneous, the expression levels are clearly distinguishable from the negative controls, and therefore, enable accurate CTC enumeration after gating. Subsequently, CTCs were gated, enumerated, and visualized as an image gallery by using CellProfiler Analyst (Fig 2B and 2C). By virtue of the pipeline, we defined different cell populations from the immunostaining of microfluidic outputs for further validation, namely, typical CTC (EpCAM/CK^+^CD45^−^), dual-positive cell (EpCAM/CK^+^CD45^+^), and the total cell.

### Clinical validation of the CTC staining and enumeration workflow

**Fig 3:**
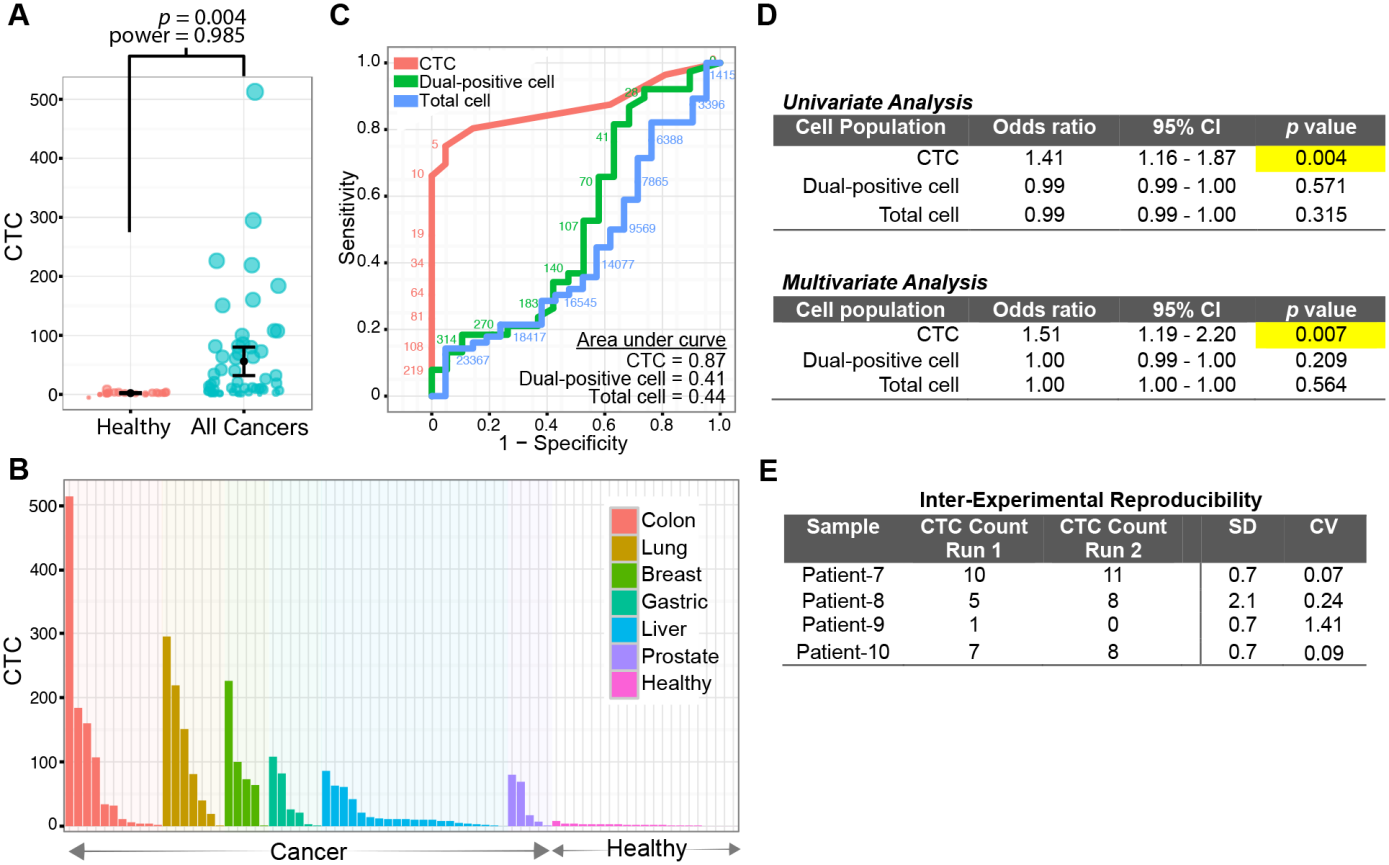
Validation analysis for the CTC enumeration pipeline. (A) CTC counts are significantly higher in cancer patients (n = 56) than those in healthy individuals (n = 21), *p* = 0.004. (B) A bar chart displaying individual CTC counts for each cancer patient and healthy donor. (C) Receiver operating characteristic (ROC) plot exhibiting the power of CTC counts in differentiating samples from cancer patients and healthy individuals. (D) Univariate and multivariate analyses testing for the correlation of the number of CTCs, dual-positive cells, and total cells with the clinical presentation of cancer (i.e. presence of cancer). (E) Inter-experimental reproducibility of CTC assays. SD: standard deviation CV: coefficient of variation (CV).

To validate our CTC staining and enumeration workflow, we conducted a pilot study involving 77 clinical samples (56 cancer patients and 21 healthy individuals) using the spiral microfluidic chip system. The numbers of typical CTCs in cancer patients are significantly higher than that in healthy individuals (*p* = 0.004, power = 0.985, two-tailed student *t* test; Fig 3A). Fig 3B depicts the CTC counts in individual patients diagnosed with six common cancer types. Apart from typical CTCs, atypical dual-positive cells were observed in the blood samples of cancer patients, but the clinical significance of this remains unclear [23]. We counted the dual-positive cells and compared their numbers with typical CTCs. The ROC curve analysis confirmed that the typical CTC count is a significant biomarker for differentiating cancer patients from normal individuals (AUC = 0.87, 95%CI = 0.79–0.95, *p* = 0.000; Fig 3C). In contrast, the number of dual-positive cells and total cells showed no differentiation value (AUC < 0.5 and *p* > 0.05). The CTC test achieved a sensitivity of 80.4% and specificity of 85.7% (cut-off value = 3.5). Additionally, as shown in Fig 3D, univariate and multivariate analysis identified a significant association between CTC number and the presence of cancer (univariate analysis: odds ratio = 1.41, *p* = 0.004, power = 1; multivariate analysis: odds ratio = 1.51, *p* = 0.007, power = 1). In contrast, no association could be found between the number of dual-positive cells or total cells with the presence of cancer (*p* > 0.05; Fig 3D). The multivariate analyses were also performed for individual cancer types (S3 Table), and the odds ratios for CTC number are consistently larger than 1. Taken together, our CTC staining protocol coupled with the illustrated enumeration pipeline was validated and shown to be highly adaptable to systematic analyses of CTCs in clinical settings.

### Cancer monitoring by CTC count

To examine the reproducibility of our CTC tests, two tubes of blood (8 ml each) were received from each of four patients. The blood samples were processed separately with different chips on two microfluidic machines. As shown in Fig 3E, the CTC enumeration results were highly reproducible with low variance. Next, we sought to explore clinical application of our CTC enumeration pipeline in serial cancer monitoring. Pre- and post-treatment blood samples from patients with hepatocellular carcinoma (n = 4) were collected for CTC counting. We observed a marked decline of CTC number in the post-treatment samples [i.e. reduction of CTC number: patient-1: 82.5%, patient-2: 66.7%, patient-3: 47.7%, and patient-4: 100%]. The decreasing trend of CTC number after treatment suggests a potential clinical utility for pharmacodynamics studies. In HCC, the EpCAM-expressing CTCs were reported as tumor-initiating cells [24], which were significantly correlated with poor prognosis of patients after tumor resection [25]. Though this evidence strongly supported the importance of EpCAM-expressing CTCs, pilot studies with larger sample size are needed to demonstrate the usefulness of CTCs in cancer monitoring. Taken together, our CTC analytical pipeline can achieve a reliable and systematic CTC assessment, which can broaden the downstream applications of CTC analysis.

### CTC enumeration is important for CTC sequencing

Apart from pharmacodynamics studies, the preceding CTC counting workflow also plays a key role for accurate somatic mutation detection in NGS study. Since the microfluidic output contains a hematopoietic cell background, the somatic mutations in CTCs are expected to occur at a low variant frequency (VF). To facilitate accurate estimation of somatic mutations, the CTC purity in the microfluidic output is used to adjust the frequency threshold and to reduce false negative error during statistical analysis. To do so, the CTC number obtained from the CTC enumeration workflow was used to estimate CTC purity (i.e. CTC number / total cell number). The CTC purity was then input into VarScan2 [18] to adjust the Fisher’s exact test for calling somatic mutations (see Methods for details).

### NGS library design and preparation

**Fig 4:**
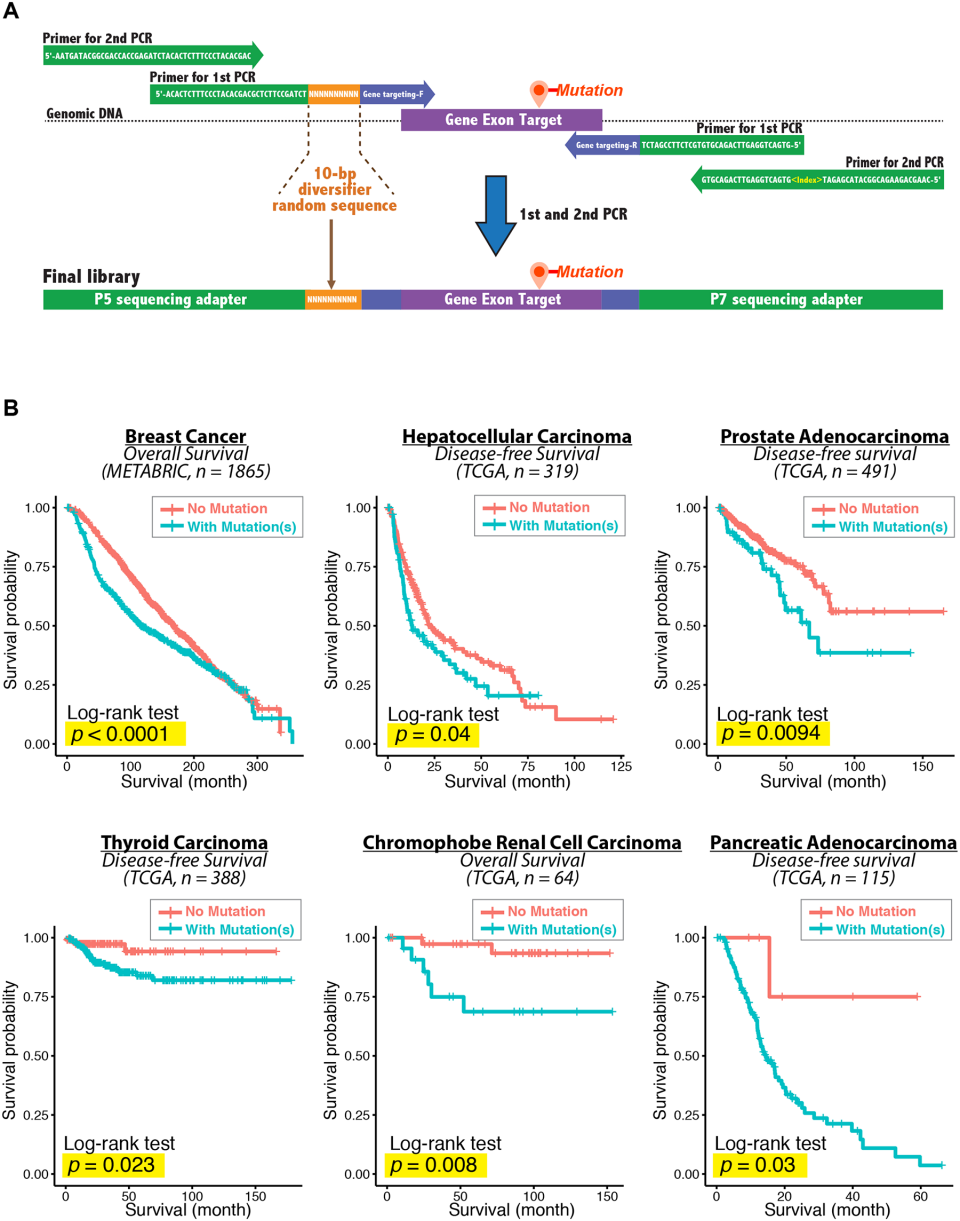
Schematic diagram of the 2-step library preparation. (A) Libraries were prepared by two PCRs. The primer pair for the first PCR contains 1) target-hybridizing sequences (blue), which bind to and amplify the exon targets; 2) a 10-bp diversifier sequence composed of random nucleotides (orange), which promotes accurate DNA cluster detection during sequencing runs; 3) a portion of P5 and P7 adapter sequences (green). The universal primer pairs for the second PCR add a full P5 and P7 sequencing adapter (green) to the libraries. Primer sequences are listed in S1 and S2 Tables. (B) Kaplan-Meier survival curves of cancer patients stratified by existence of mutations in their tumor tissues. Patients were grouped into “No mutation” or “With mutation(s)” for analysis, according to their mutation status of *NRAS*, *ESR1*, *EGFR*, *KRAS*, *BRAF and TP53* genes. The log-rank test *p* values comparing two survival curves are shown in each plot.

The preparation of NGS library is straightforward; it simply involves two PCR steps for library preparation (Fig 4A). The resulting amplicon libraries contain 1) P5 and P7 adapters for binding onto the flowcell; 2) a 10-bp diversifier sequence; and 3) targeted exon region. The 10-bp diversifier sequence composes of random Ns and is essential for enhancing the library diversity to achieve precise DNA cluster identification during the sequencing. On the flowcell of the Illumina sequencing system, each DNA cluster is a single sequencing read, which needs to be precisely detected for high-quality sequencing outputs. In the Illumina MiSeq, NextSeq, and HiSeq platforms, the success of DNA cluster identification and mapping largely relies on the sequence diversity of the library during the first five sequencing cycles. However, the amplicon library generally is low in sequence diversity, which can cause ambiguous cluster detection, thereby resulting in low-quality reads and data loss after sequence filtering. To increase sequence diversity for accurate cluster identification, we introduced a sequence composed of random Ns in the amplicon libraries.

In our sequencing workflow, we sought to sequence clinically relevant cancer genes harboring pathogenic mutations and/or clinically actionable mutations. The targeting genes in our library design include *NRAS*, *ESR1*, *EGFR*, *KRAS*, *BRAF*, and *TP53*. We utilized TCGA and METABRIC datasets to explore the clinical relevance of this gene panel. KM survival analysis showed significant associations (log-rank *p* value < 0.05) between mutation occurrence and the patient survival in breast cancer (n = 1865), liver cancer (n = 319), prostate cancer (n = 491), thyroid cancer (n = 388), kidney cancer (n = 64), and pancreatic cancer (n = 115) (Fig 4B). Cancer patients with mutation(s) in the gene panel were associated with poor overall survival or disease-free survival. We envision that sequencing this gene panel in CTCs may also provide important information in both academic and clinical settings.

**Fig 5:**
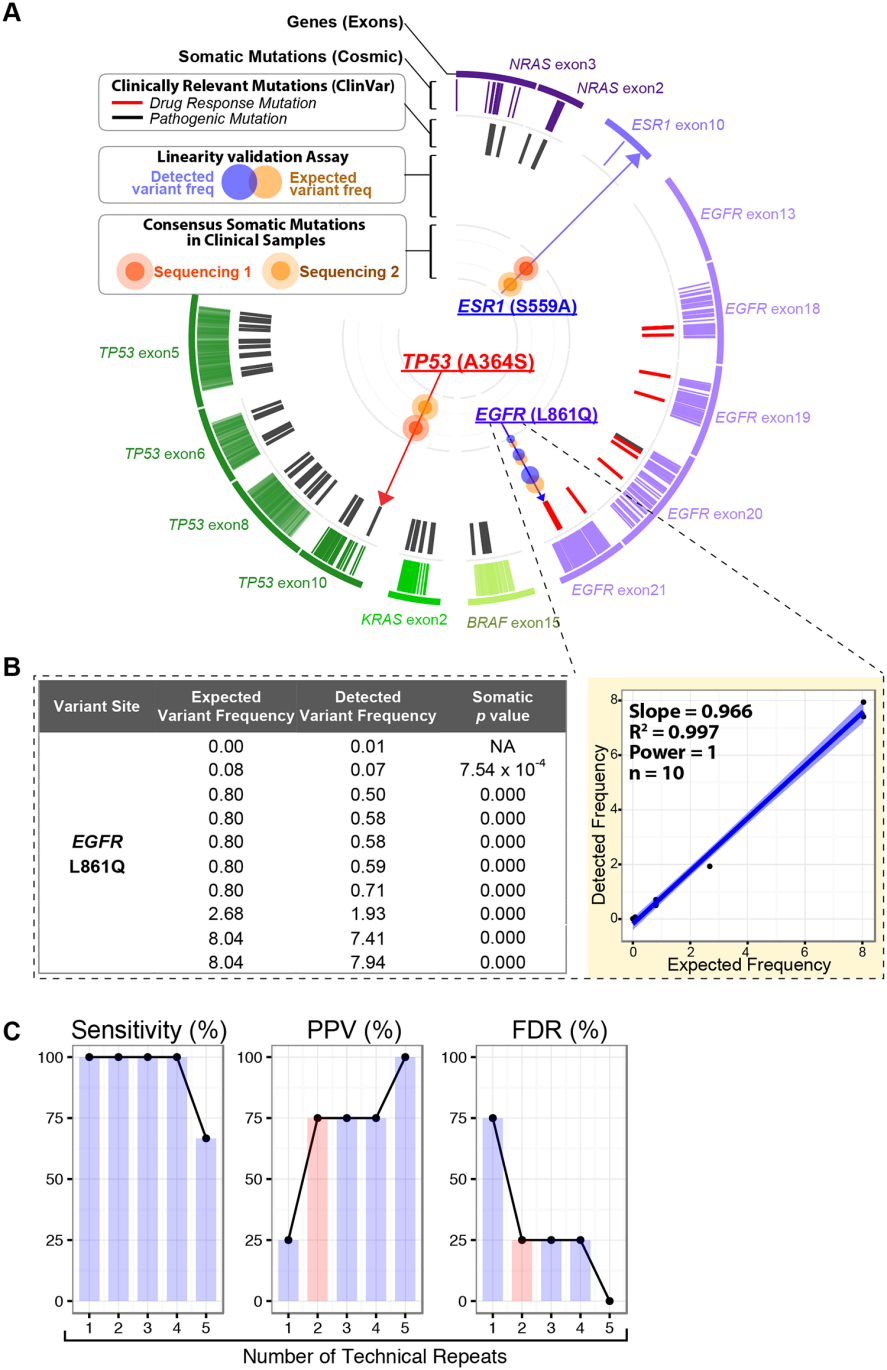
Sequencing target regions and the sequencing results. (A) Descriptions from outer track to inner track are as follows: Track 1 (target genes and exons sequenced); Track 2 (somatic mutations): The bars indicate somatic mutations reported in the COSMIC database; Track 3 (clinically-relevant mutations): The bars represent clinically relevant mutations reported in the ClinVar database; Track 4 (validation assay): The interlaced dots indicate three representative sequencing results of simulated CTC samples. The size of the dots is proportional to the frequency. See (B) for the correlation between frequencies. Track 5 (consensus somatic mutations detected in the clinical samples): library preparation and sequencing of CTC microfluidic outputs were performed in technical duplicates. Consensus mutations are shown in dots. (B) Results of dilution sequencing assay for linearity validation. The relationship between the expected and the detected frequency is shown. (C) Bar charts showing the change in sensitivity, positive prediction value (PPV), and false discovery rate (FDR), after increasing the number of technical replicates. An obvious improvement of PPV and FDR was noted and indicated as pink bars.

To demonstrate the sequencing workflow for the gene panel, we designed primers flanking the target exons of *NRAS*, *ESR1*, *EGFR*, *KRAS*, *BRAF*, and *TP53* genes, which harbor clinically relevant somatic mutations as recorded in the Catalogue Of Somatic Mutations (COSMIC) and ClinVar databases. Tracks 1 to 3 in Fig 5A depict the sequencing gene targets and the associated mutations (pathogenic or drug-response associated mutations) reported in the COSMIC and ClinVar databases.

### Validation of sequencing performance

Next we benchmarked our targeted sequencing workflow by sequencing a series of simulated microfluidic outputs. To mimic hematopoietic cell background, we applied a widely used reference DNA control (NA12878), which is a gold standard benchmark validated by the NIST-led Genome in a Bottle Consortium. To mimic CTCs, we used DNA from an esophageal cancer cell line (KYSE 270), which contains a clinically relevant *EGFR* L861Q mutation [26, 27], as recorded in the COSMIC database.

To evaluate whether the detected variant frequency (VF) from the sequencing workflow is linearly correlated with the expected VF, we conducted a linearity validation assay using a series of simulated CTC microfluidic outputs by spiking different percentages of KYSE270 DNA (mimicking CTCs) into a background of NA12878 DNA (mimicking hematopoietic cell background). The expected VFs from simulated CTC samples were 0%, 0.08%, 0.8%, 2.68%, and 8% for *EGFR* L861Q mutation (Fig 5B). Remarkably, our sequencing workflow showed a high linearity and correlation coefficient between the expected and the detected VF (n = 10, R^2^ = 0.997, slope = 0.966, power = 1; Fig 5B). In addition, the *EGFR* L861Q mutation can be detected even down to 0.08% expected VF (somatic *p* value = 7.54 x 10^−4^), which indicated a high detection sensitivity for the sequencing workflow (Fig 5B). Furthermore, by analyzing the simulated CTC samples (n = 5, 0.8% VF), we observed that the performance of mutation identification can be improved by increasing the number of technical replications (Fig 5C). Particularly, in technical duplication, when consensus mutations across the sequencing results were called, there is an obvious elevation of positive prediction value (PPV) (from 25% to 75%) and a corresponding decline of false discovery rate (FDR) (Fig 5C), whereas the sensitivity remains steadily high (100%). Parenthetically, the sequencing performance may also be improved by testing various bioinformatics tools [28], but this is beyond the scope of present study.

### Sequencing clinical CTC samples

By virtue of our sequencing workflow, we sequenced microfluidic outputs from two patients diagnosed with hepatocellular carcinoma. The patients’ WBCs were used as a reference control for bioinformatics analysis. Library preparation and sequencing of CTC microfluidic outputs were performed in technical duplicates. Consensus mutations across the technical repeats, with significant somatic *p* values, were called for further analysis. We found a *TP53* somatic missense mutation (A364S) and an *ESR1* missense mutation (S559A) in CTC samples (Fig 5A and Table 1). *In silico* prediction suggested a damaging effect conferred by this *TP53* A364S mutation (Table 1). It is noteworthy that the missense mutation on A364 site was reported in ovarian cancer in the COSMIC database (mutation ID: COSM46361). On the other hand, *ESR1* was linked to the susceptibility to HCC [29] and was suggested to be a tumor suppressor gene in HCC [30]. Mutation frequency of *ESR1* is 1.6% (6/366 sequenced cases) in the TCGA provisional data set of HCC. Taken together, we demonstrated a straightforward amplicon-based targeted sequencing workflow to gain insight into the genetic discovery of CTCs.

**Table 1:** Sequencing results for the CTC microfluidic outputs from patients.

Sample	CTC number	Genomic coordinate	Mutation	Somatic *p* value in technical repeat (coverage)	Predictor | Score | Prediction
***Patient-12***	10	Chr17: 7573937	A364S	***Run 1***	FATHMM score | -5.49 | Deleterious RadialSVM score | 0.76 | Damaging LR score | 0.91 | Damaging
				*p* = 1.8 x 10^−7^(448952)	
				***Run 2***	
				*p* = 1.9 x 10^−3^(335959)	
***Patient-19***	45	Chr6: 152419988	S559A	***Run 1***	PolyPhen 2 HDIV | 0.95 | Possibly damaging PolyPhen 2 HVAR | 0.80 | Possibly damaging MutationTaster | 0.78 | Disease-causing
				*p* = 7.9 x 10^−4^ (57352)	
				***Run 2***	
				*p* = 0.023 (62598)	

## Conclusion

In conclusion, this report demonstrates a highly adaptable workflow for CTC staining, enumeration, and targeted sequencing. A systematic workflow for CTC enumeration and genetic analysis can bridge the technical gap between the advancement of CTC microfluidic enrichment and systematic investigation of CTCs. An integrated pipeline of the fascinating microfluidic advances and the illustrated workflows can pave the way to better define the biology of CTCs and explore their clinical applications in cancer monitoring and personalized medicine.

## Supporting information

S1 Table Primers used in the 1st PCR step of library preparation. (DOCX)

S2 Table Primers used in the 2nd PCR step of library preparation. (DOCX)

S3 Table Multivariate analyses of individual cancer types. (DOCX)
