# Supplementary material for: Succinct workflows for circulating tumor cells after enrichment: From systematic counting to mutational profiling
Source: PLoS One. 2017 May 8;12(5):e0177276. doi: 10.1371/journal.pone.0177276 (PMC5421802; doi:10.1371/journal.pone.0177276)
Supplement: S1 Table — (DOCX) [file pone.0177276.s001.docx]

**S1 Table: Primers used in the 1^st^ PCR step of library preparation**

| Gene | Exon | Primer Name | Primer sequence for 1st PCR | Size of PCR product |
| --- | --- | --- | --- | --- |
| EGFR | 21 | EGFRExon21_F | ACACTCTTTCCCTACACgACgCTCTTCCgATCTNNNNNNNNNNCCTCACAgCAgggTCTTCTC | 205bp |
|  |  | EGFRExon21_R | gTgACTggAgTTCAgACgTgTgCTCTTCCgATCTTgACCTAAAgCCACCTCCTTAC |  |
| TP53 | 8 | TP53Exon8_F | ACACTCTTTCCCTACACgACgCTCTTCCgATCTNNNNNNNNNNgCCTCTTgCTTCTCTTTTCC | 200bp |
|  |  | TP53Exon8_R | gTgACTggAgTTCAgACgTgTgCTCTTCCgATCTCTCCTCCACCgCTTCTTgTC |  |
| TP53 | 7 | TP53Exon7_F | ACACTCTTTCCCTACACgACgCTCTTCCgATCTNNNNNNNNNNCTTgggCCTgTgTTATCTCC | 171bp |
|  |  | TP53Exon7_R | gTgACTggAgTTCAgACgTgTgCTCTTCCgATCTCAgCAggCCAgTgTgCAg |  |
| TP53 | 5 | TP53Exon5_F | ACACTCTTTCCCTACACgACgCTCTTCCgATCTNNNNNNNNNNCTgTCTCCTTCCTCTTCCTACAg | 240bp |
|  |  | TP53Exon5_R | gTgACTggAgTTCAgACgTgTgCTCTTCCgATCTAgCCCTgTCgTCTCTCCAg |  |
| TP53 | 6 | TP53Exon6_F | ACACTCTTTCCCTACACgACgCTCTTCCgATCTNNNNNNNNNNCCCCAggCCTCTgATTCC | 167bp |
|  |  | TP53Exon6_R | gTgACTggAgTTCAgACgTgTgCTCTTCCgATCTCCCCAgTTgCAAACCAgAC |  |
| TP53 | 10 | TP53Exon10_F | ACACTCTTTCCCTACACgACgCTCTTCCgATCTNNNNNNNNNNCCCCTCCTCTgTTgCTgC | 156bp |
|  |  | TP53Exon10_R | gTgACTggAgTTCAgACgTgTgCTCTTCCgATCTAggAAggggCTgAggTCAC |  |
| BRAF | 15 | BRAFExon15_F | ACACTCTTTCCCTACACgACgCTCTTCCgATCTNNNNNNNNNNTgTTTTCCTTTACTTACTACACCTCA | 198bp |
|  |  | BRAFExon15_R | gTgACTggAgTTCAgACgTgTgCTCTTCCgATCTAgCATCTCAgggCCAAAAAT |  |
| EGFR | 13 | EGFRExon13_F | ACACTCTTTCCCTACACgACgCTCTTCCgATCTNNNNNNNNNNCACTgACTgCTgTgACCCAC | 196bp |
|  |  | EGFRExon13_R | gTgACTggAgTTCAgACgTgTgCTCTTCCgATCTCgCAAggggATTAAAgAAATAAC |  |
| EGFR | 18 | EGFRExon18_F | ACACTCTTTCCCTACACgACgCTCTTCCgATCTNNNNNNNNNNgCTgAggTgACCCTTgTCTC | 216bp |
|  |  | EGFRExon18_R | gTgACTggAgTTCAgACgTgTgCTCTTCCgATCTCAgACCATgAgAggCCCTg |  |
| EGFR | 19 | EGFRExon19_F | ACACTCTTTCCCTACACgACgCTCTTCCgATCTNNNNNNNNNNAATTgCCAgTTAACgTCTTCC | 198bp |
|  |  | EGFRExon19_R | gTgACTggAgTTCAgACgTgTgCTCTTCCgATCTgAgAAAAggTgggCCTgAg |  |
| EGFR | 20 | EGFRExon20_F | ACACTCTTTCCCTACACgACgCTCTTCCgATCTNNNNNNNNNNgACgTgCCTCTCCCTCC | 235bp |
|  |  | EGFRExon20_R | gTgACTggAgTTCAgACgTgTgCTCTTCCgATCTTCCCCgTATCTCCCTTCC |  |
| ESR1 | 10 | ESR1Exon10_F | ACACTCTTTCCCTACACgACgCTCTTCCgATCTNNNNNNNNNNCCTTTCTgTgTCTTCCCACC | 176bp |
|  |  | ESR1Exon10_R | gTgACTggAgTTCAgACgTgTgCTCTTCCgATCTCAAgTggCTTTggTCCgTC |  |
| KRAS | 2 | KRASExon2_F | ACACTCTTTCCCTACACgACgCTCTTCCgATCTNNNNNNNNNNgCCTgCTgAAAATgACTgAA | 167bp |
|  |  | KRASExon2_R | gTgACTggAgTTCAgACgTgTgCTCTTCCgATCTAgAATggTCCTgCACCAgTAA |  |
| NRAS | 3 | NRASExon3_F | ACACTCTTTCCCTACACgACgCTCTTCCgATCTNNNNNNNNNNCACCCCCAggATTCTTACAg | 173bp |
|  |  | NRASExon3_R | gTgACTggAgTTCAgACgTgTgCTCTTCCgATCTTCCgCAAATgACTTgCTATT |  |
| NRAS | 2 | NRASExon2_F | ACACTCTTTCCCTACACgACgCTCTTCCgATCTNNNNNNNNNNggTTTCCAACAggTTCTTgC | 158bp |
|  |  | NRASExon2_R | gTgACTggAgTTCAgACgTgTgCTCTTCCgATCTgCTACCACTgggCCTCAC |  |
