# Supplementary material for: Succinct workflows for circulating tumor cells after enrichment: From systematic counting to mutational profiling
Source: PLoS One. 2017 May 8;12(5):e0177276. doi: 10.1371/journal.pone.0177276 (PMC5421802; doi:10.1371/journal.pone.0177276)
Supplement: S2 Table — (DOCX) [file pone.0177276.s002.docx]

**S2 Table: Primers used in the 2^nd^ PCR step of library preparation**

| Primer Name | Primer sequence for 2nd PCR |
| --- | --- |
| Index1 primer | CAAgCAgAAgACggCATACgAgATCgTgATgTgACTggAgTTCAgACgTg |
| Index3 primer | CAAgCAgAAgACggCATACgAgATgCCTAAgTgACTggAgTTCAgACgTg |
| Index9 primer | CAAgCAgAAgACggCATACgAgATCTgATCgTgACTggAgTTCAgACgTg |
| Index10 primer | CAAgCAgAAgACggCATACgAgATAAgCTAgTgACTggAgTTCAgACgTg |
| Index11 primer | CAAgCAgAAgACggCATACgAgATgTAgCCgTgACTggAgTTCAgACgTg |
| Universal primer | AATgATACggCgACCACCgAgATCTACACTCTTTCCCTACACgAC |
