# Supplementary material for: Succinct workflows for circulating tumor cells after enrichment: From systematic counting to mutational profiling
Source: PLoS One. 2017 May 8;12(5):e0177276. doi: 10.1371/journal.pone.0177276 (PMC5421802; doi:10.1371/journal.pone.0177276)
Supplement: S3 Table — (DOCX) [file pone.0177276.s003.docx]

| **S3 Table: Multivariate analyses of individual cancer types** | | |
| --- | --- | --- |
| ***(Breast cancer, n = 5)*** | |  |
| ***Cell population*** | ***Odds ratio*** | ***p* value** |
| CTC | 1.14 | 0.36 |
| TCC | 0.99 | 0.41 |
| Double positive | 0.99 | 0.55 |

***(Colorectal cancer, n = 11)***

| ***Cell population*** | ***Odds ratio*** | ***p value*** |
| --- | --- | --- |
| CTC | 2.11 | 0.08^†^ |
| TCC | 0.99 | 0.97 |
| Double positive | 0.99 | 0.25 |

***(Lung cancer, n = 7)***

| ***Cell population*** | ***Odds ratio*** | ***p value*** |
| --- | --- | --- |
| CTC | 2.58 | 0.21 |
| TCC | 0.99 | 0.37 |
| Double positive | 0.97 | 0.39 |

***(Gastric cancer, n = 6)***

| ***Cell population*** | ***Odds ratio*** | ***p value*** |
| --- | --- | --- |
| CTC | 1.40 | 0.04* |
| TCC | 0.99 | 0.96 |
| Double positive | 1.00 | 0.09 |

***(Hepatocellular carcinoma, n = 22)***

| ***Cell population*** | ***Odds ratio*** | ***p value*** |
| --- | --- | --- |
| CTC | 1.64 | 0.01* |
| TCC | 0.99 | 0.22 |
| Double positive | 0.99 | 0.50 |

***(Prostate cancer, n = 5)***

| ***Cell population*** | ***Odds ratio*** | ***p value*** |
| --- | --- | --- |
| CTC | 1.86 | 0.16 |
| TCC | 0.99 | 0.56 |
| Double positive | 0.99 | 0.39 |

Abbreviation: TCC, total cell count

^†^ Marginally significant

^*^ Statistically significant
